# Hydroxyurea down-regulates *BCL11A, KLF*-*1* and *MYB* through miRNA-mediated actions to induce γ-globin expression: implications for new therapeutic approaches of sickle cell disease

**DOI:** 10.1186/s40169-016-0092-7

**Published:** 2016-04-07

**Authors:** Gift Dineo Pule, Shaheen Mowla, Nicolas Novitzky, Ambroise Wonkam

**Affiliations:** Division of Human Genetics, Faculty of Health Sciences, University of Cape Town, Anzio Road, Observatory, Cape Town, 7925 Republic of South Africa; Division of Hematology, Department of Clinical Laboratory Sciences, Faculty of Health Sciences, University of Cape Town, Cape Town, Republic of South Africa

**Keywords:** Hydroxyurea, γ-Globin expression, Human erythroid, *BCL11A*, *KLF*-*1*, *MYB*, miRNAs-mediated actions

## Abstract

**Background:**

The major therapeutic benefit of hydroxyurea, the only FDA-approved pharmacologic treatment for sickle cell disease (SCD), is directly related to fetal hemoglobin (HbF) production that leads to significant reduction of morbidity and mortality. However, potential adverse effects such as infertility, susceptibility to infections, or teratogenic effect have been subject of concerns. Therefore, understanding HU molecular mechanisms of action, could lead to alternative therapeutic agents to increase HbF with less toxicity. This paper investigated whether HU-induced HbF could operate through post-transcriptional miRNAs regulation of *BCL11A, KLF*-*1* and *MYB,* potent negative regulators of HbF. Both ex vivo differentiated primary erythroid cells from seven unrelated individuals, and K562 cells were treated with hydroxyurea (100 μM) and changes in *BCL11A*, *KLF*-*1*, *GATA*-*1*, *MYB,* β- and γ-globin gene expression were investigated. To explore potential mechanisms of post-transcriptional regulation, changes in expression of seven targeted miRNAs, previously associated with basal γ-globin expression were examined using miScript primer assays. In addition, K562 cells were transfected with miScript miRNA inhibitors/anti-miRNAs followed by Western Blot analysis to assess the effect on HbF protein levels. Direct interaction between miRNAs and the *MYB* 3′-untranslated region (UTR) was also investigated by a dual-luciferase reporter assays.

**Results:**

Down-regulation of *BCL11A* and *MYB* was associated with a sevenfold increase in γ-globin expression in both primary and K562 cells (p < 0.003). Similarly, *KLF*-*1* was down-regulated in both cell models, corresponding to the repressed expression of *BCL11A* and β-globin gene (p < 0.04). HU induced differential expression of all miRNAs in both cell models, particularly miR-15a, miR-16, miR-26b and miR-151-3p. An HU-induced miRNAs-mediated mechanism of HbF regulation was illustrated with the inhibition of miR-26b and -151-3p resulting in reduced HbF protein levels. There was direct interaction between miR-26b with the *MYB* 3′-untranslated region (UTR).

**Conclusions:**

These experiments have shown the association between critical regulators of γ-globin expression (*MYB*, *BCL11A* and *KLF*-*1*) and specific miRNAs; in response to HU, and demonstrated a mechanism of HbF production through HU-induced miRNAs inhibition of *MYB*. The role of miRNAs-mediated post-transcriptional regulation of HbF provides potential targets for new treatments of SCD that may minimize alterations to the cellular transcriptome.

**Electronic supplementary material:**

The online version of this article (doi:10.1186/s40169-016-0092-7) contains supplementary material, which is available to authorized users.

## Background

Sickle cell disease (SCD) occupies a prominent place in human genetics, as the paradigmatic example of a monogenic disorder, that was described over 100 years ago, and in 1949, it became the first human disease to be deciphered at the molecular level [1]. SCD is a monogenic, hematological and multi-organ disorder associated acute and chronic illness, and progressive organ damage [2]. The disease is due to a single point mutation (Glu6Val) that causes polymerization of the mutant hemoglobin (Hb) S, resulting in sickling of erythrocytes. Inflammation, hemolysis, microvascular obstruction and organ damage characterize the clinical expression of SCD, which is highly variable in individual patients [3]. Sub-Saharan Africa (SSA) has the highest disease burden with approximately 305,800 affected new-borns per year, accounting for 0.74 % of all births in the region (Modell et al. 2008) and 80 % of annual global affected child births [4]. Despite this high incidence, the life-saving public health programs have not been implemented in most SSA countries often associated with limited medical resources and infrastructures. As a consequence, neonatal and childhood mortality due to SCD remains high and estimates suggest that without intervention, up to 90 % of affected children in SSA die by age five from SCD [4, 5]. Nevertheless, SCD patients manifest with wide varying degrees of severity. In combination with environmental factors, several genomic loci, influence the clinical course of SCD. Indeed, despite the fact that there are several key phenotypes of SCD (anemia, stroke, infections), fetal hemoglobin (HbF) has emerged as a central disease modifier [3], that is amenable to therapeutic manipulation [6]. Genetic variants at three principal loci, *BCL11A*, *HBS1L*-*MYB* and *HBB* cluster account for 10–20 % of HbF variation [7–9]; among SCD patients in USA and Brazil [10], Tanzania [5] and Cameroon [11]. Currently, hydroxyurea (HU) is the only FDA-approved pharmacologic treatment for the induction of HbF in patients with SCD. The major HU benefit is directly related to its HbF-producing effect [12, 13] that leads to significant reduction of pain, acute chest episodes, mortality and the need for blood transfusions [14–17]. HU has also been associated with clinical drift, where physicians use the drug for related complications of SCD such as stroke prevention, priapism and pulmonary hypertension [18]. However potential short and long term adverse effects such as infertility [18–20], susceptibility to infections [21–24], potential teratogenic effect [25, 26], have also been associated with HU. The fear of such side-effects has been a subject of concern to some professionals [27, 28], parents as well as patients [29–33] and a potential barrier to compliance in some settings [34, 35]. As a consequence, HU is still underutilized [29, 30]. It is then urgent to fully understand HU molecular mechanisms of action, in order to explore alternative and potential less toxic and more acceptable agents that could equally increase the level of HbF.

Several other HU-mediated mechanisms of disease amelioration have been reported including production of nitric oxide, regulation of soluble guanylyl cyclase, cyclic adenosine and guanosine monophosphate [36, 37] as well as erythropoietic stress response [38]. Furthermore, various signalling pathways have been implicated in HU-mediated fetal hemoglobin (HbF) induction such as the Giα/JNK/Jun [39]; p38/MAPK/CREB1 [40]; cAMP-mediated response [41, 42]; erythropoietin (EPO)-induced activation of the ERK-1/ERK-2 MAPK [43]; histone deacetylase (HDAC) and DNA methyl-transferase (DNMT) inhibitor-mediated epigenetic modification of γ-globin expression. Despite this, a complete understanding of HU-mediated HbF production remains incomplete.

Post-transcriptional regulation of γ-globin expression through micro RNAs (miRNAs) has been shown to play an important role in HU-mediated HbF induction as HU causes differential expression of a suite of miRNAs associated with basal and γ-globin expression at maximum tolerated dose (MTD) in SCD patients [44, 45]. Likewise, DNA methylation has been significantly associated with baseline HbF [38, 46, 47] but provided no substantial explanation for HbF induction in response to HU. Small non-coding RNAs, particularly miRNAs, however, have emerged as powerful regulators and modifiers of gene expression through inhibition of mRNA translation [48], which has implications for hematopoiesis and erythropoiesis [49, 50], particularly in the distinct miRNA expression patterns in SCD patients [51] and the severity of anaemia [52]. Moreover, a few reports have specifically implicated the miRNAs in the regulation of HbF [44, 53, 54].

This study has further investigated whether the induction of HbF by HU treatment could operate through post-transcriptional regulation of *BCL11A, KLF*-*1* and *MYB*, by analysing changes in expression of select miRNAs in erythroid cells derived from umbilical cord blood CD34^+^ hematopoietic stem cells and the K562 cell line.

## Results

### CD34^+^ hematopoietic cells

**Fig. 1:**
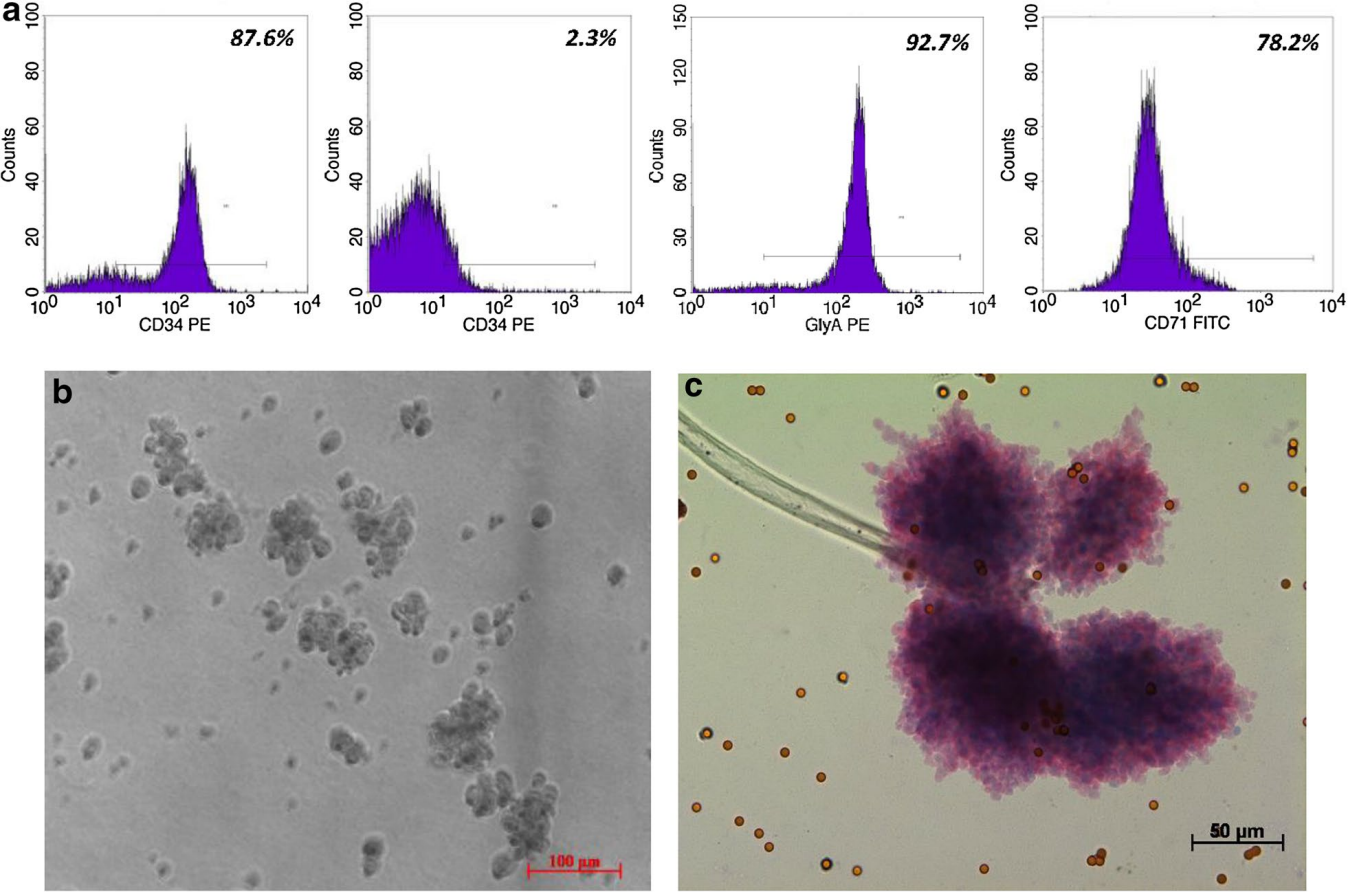
Flow cytometry showing differentiation of CD34^+^ cells to erythroid progenitors (**a**) and the cluster-like basophilic erythroblast morphology of erythroid cells on day 7 of differentiation (**b**) and the colony-forming hematopoietic cell units (CFU-E) (**c**). CD34^+^ hematopoietic stem cells were purified from umbilical cord blood mononuclear cells using Dynabead magnetic separation to a purity of 87.6 %. After the 15 day single-phase ex vivo expansion and differentiation, the cell population was 92.7 and 78.2 % CD235a^+^ and CD71^+^, respectively with minimal CD34 positivity. This confirmed our differentiation and provided erythroid cells for down-stream experiments (**a**). During the expansion and differentiation of HSCs to erythroid cells, the cells formed cluster-like colonies before separating into single cells in suspension, which was typical of terminal differentiation (**b**). The hematopoietic colony formation assay was performed to confirm the stem-like behaviour of the Dyna-bead selected CD34^+^ HSCs prior to expansion and differentiation. The colony-forming unit- granulocyte, erythroid, monocyte, megakaryocyte (CFU-GEMM) semi-solid cultures were grown for 14 days and stained for colony counting (**c**)

The average volume of umbilical cord blood collected was 108 ml (±25 ml) with a 1.3 % (±0.5 %) yield of CD34^+^ cells at an average 87 % (±6 %) percentage purity of the cell population after Dynabead magnetic separation technology (Fig. 1a). The CFU-GEMMs also produced several burst-forming unit-erythroid cells (BFU-E) and erythroid clusters typically formed during expansion (Fig. 1b).

### Ex-vivo differentiation of CD34^+^ cells

CD34^+^ cells were cultured using an ex vivo single-phase expansion and differentiation protocol and all primary CD34^+^ primary lines were successfully expanded (mean fold expansion 4.2; ± 2.7) and differentiated into erythroid progenitors with average expression of 84 % (±3 %) and 83 % (±8 %) for CD235a and CD71, respectively (Fig. 1a). The first half of the expansion and differentiation was characterized by cluster-like colonies of erythroid cells and sparse loose-lying cells (Fig. 1b, c). During early differentiation (days 1–3) large blasts were observed in culture followed by proerythroblast morphology around day 5. The morphology typical of basophilic erythroblasts was observed on days 7–8 (Fig. 1b) and on day 15, the majority of the cells had ortho/polychromatophilic morphology, which was confirmed by the expression of cell-surface markers CD71 and CD235a (Additional file 1: Figure S1), at which point differentiation was halted and cells treated with HU for various analyses.

### WST-1 cell proliferation assay

To determine the optimal concentration and exposure time to HU, K562 cells (8 × 10^4^ cells) were treated with varying doses of HU and for 2, 4, 6, 12 and 24 h. Six hour (6) exposure time and 100 µM were determined to be optimal as there was minimal initial cytotoxic effect and sufficient cellular metabolic activity to alter gene expression in response to HU (Additional file 2: Figure S2).

### Hydroxyurea induces γ-globin expression in both erythroid and K562 cells

**Fig. 2:**
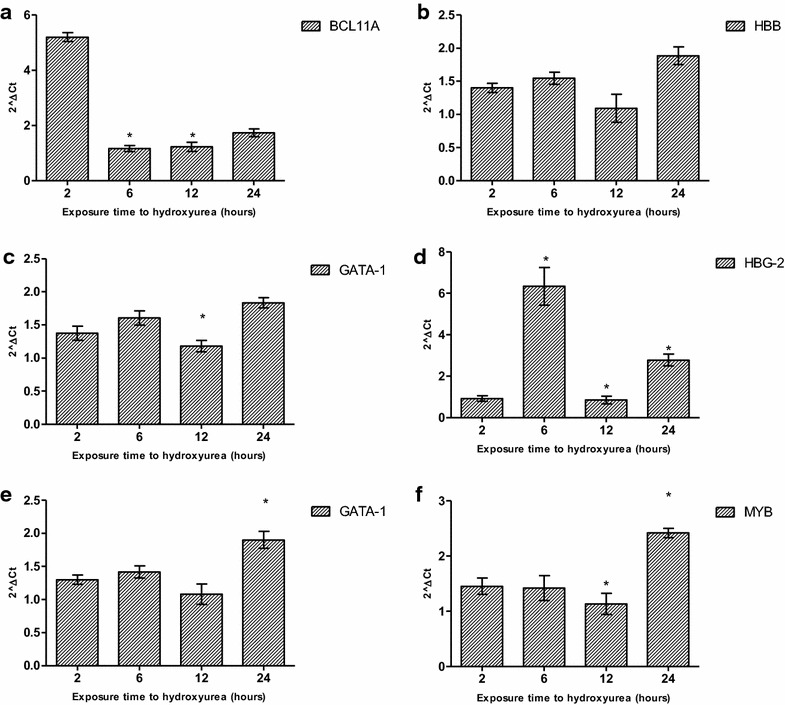
Time-dependent gene expression changes in K562 cells treated with hydroxyurea Hydroxyurea induced inverse time-dependent sigmoidal expression of *BCL11A* and *HBG*-1 (HbF) with the highest fold increase in HbF corresponding to the lowest *BCL11A* expression between 6 and 12 h after HU treatment. *KLF*-1, an activator of *BCL11A*, also followed a similar trend, lowest after 12 h of HU treatment. *GATA*-1 was also down-regulated at the same time point as well as the negative regulator, *MYB*. * Significant difference, p < 0.05

Erythroid progenitors from seven unrelated individuals and the K562 cell line were successfully analysed for differential gene expression in response to HU treatment, with three technical and independent qPCR experimental repeats for K562 cells and one experimental repeat for ex vivo derived erythroid cells due to the limited number of cells. Subsequently, four of the ex vivo derived lines were analysed for corresponding miRNA expression. HU treatment of K562 cells showed a sigmoidal pattern of expression in *BCL11A* transcription with an apparent down-regulation at 6 h, an expression pattern inverse to the γ-globin that showed a sevenfold upregulation at the same HU exposure time (Fig. 2). *KLF*-*1*, a critical activator of *BCL11A* [55], and *MYB*, a potent activator of *KLF*-*1* [56, 57] and negative regulator of γ-globin [58] had similar expression patterns and were significantly down-regulated at 12 h (p < 0.03). β-Globin and *GATA*-*1* expression remained largely unaffected by HU treatment with the exception of a slight down-regulation also after 12 h treatment.

**Fig. 3:**
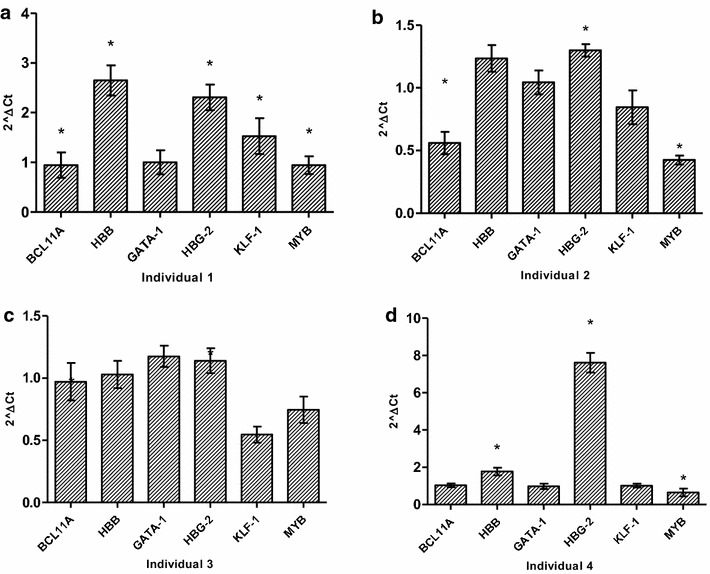
Hydroxyurea-induced gene expression changes in ex vivo derived erythroid cells. The gene expression profiles of primary erythroid lines after HU treatment for 6 h. Although the expected inter-individual variation in expression was observed, there were several common trends such the low *BCL11A*, *KLF*-1 and *MYB*; and significant upregulation of HbF expression. *HBB* (β-globin) expression was always associated with *BCL11A*, which is expected given the role of this transcription factor in the “fetal switch”. * Significant difference, p < 0.05

The gene expression pattern in the primary erythroid lines was largely similar to that in K562 cells with an inverse relationship between γ-globin expression and its critical regulators; *BCL11A*, *KLF*-*1* and *MYB*. Some primary lines had a sevenfold (p < 0.003) up-regulation of γ-globin expression 6 h after HU treatment associated with the down-regulation of *MYB* (p < 0.04) and *BCL11A* expression (Fig. 3). Gene expression analysis was done after successful ex vivo differentiation of primary erythroid cells and HU treatment for 24 h.

### Hydroxyurea up-regulates miRNAs associated with inhibition of *BCL11A* and *MYB*

**Fig. 4:**
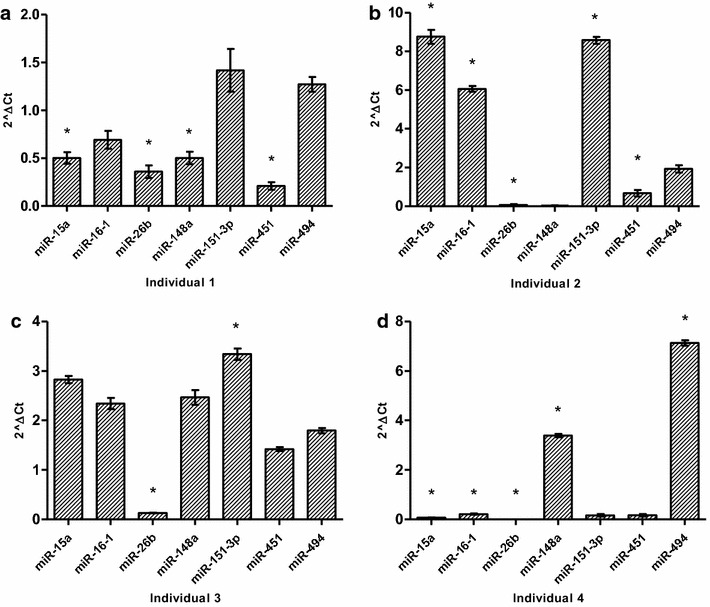
Hydroxyurea induces differential expression of miRNAs associated with *BCL11A* and *MYB* repression in erythroid cells. Treatment of primary cells with HU causes differential regulation of miRNAs as early as 6 h post treatment. There is inter-individual variation in expression however, miR-26b was significantly down-regulated in all but one primary lines. And conversely, miR-451 is down-regulated in all but 1 of the erythroid lines. HU induces variation in the expression of miRNAs associated with key regulators of HbF. * Significant difference, p < 0.05

In the primary lines analysed for miRNAs expression 6 h post treatment, there was inter-individual variation in expression however, miR-26b was significantly down-regulated in all but one primary line (Fig. 4). HU treatment also caused sigmoidal time-dependent changes in miRNAs expression in K562 cells (Additional file 3: Figure S3), with significant up-regulation of miR-15a and miR-16-1 at 6 h (*p* value 0.027 and 0.002, respectively), which are known inhibitors of *MYB* [54]. MiR-151-3p and miR-451 were also significantly up-regulated at 6 h post treatment (0.041 and 0.042, respectively) and although not statistically significant, miR-494 had a twofold increase. All miRNAs were down-regulated 12 h after treatment and returned to baseline after 24 h.

### MicroRNA inhibition

**Fig. 5:**
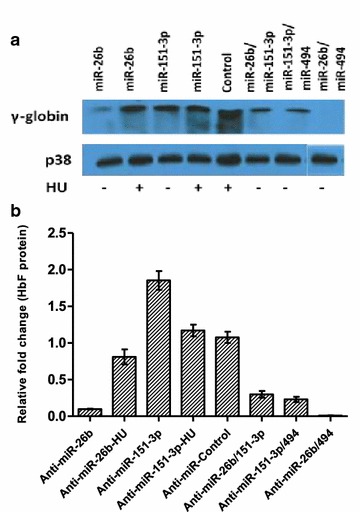
Inhibition of candidate miRNAs down-regulates HbF in K562 cells. The effect of anti-miRNAs (5 mM) on HbF protein levels in K562 cells (western blot analysis—**a**; western blot densitometric illustration—**b**). Inhibition of miR-26b and miR-151-3p results in decreased HbF levels in comparison to the control anti-miRNA. HbF expression is partially recovered with HU treatment. All anti-miRNAs co-transfections result in reduced HbF levels, particularly co-transfection of anti-miR-26b and anti-miR-494 resulting in near-complete transient knock-down of HbF

The inhibition of miR-26b and miR-151-3p resulted in a significant decrease in γ-globin expression (Fig. 5), which could be partially rescued when cells were treated with HU. This suggests that most miRNAs target negative regulators of HbF as their inhibition causes up-regulation of γ-globin expression. At a higher concentration (25 nM), all miRNAs down-regulated HbF (Additional file 4: Figure S4). Cells were co-transfected with select pairing of miRNAs in order to investigate the combinatorial effect of anti-miRNAs. The effect of miR-26b/miR-151-3p and miR-151-3p/miR-494 were comparable in the decrease of HbF. Although the inhibition of miR-494 had no apparent effect on HbF, co-transfection with miR-26b resulted in near-complete transient knock-down of HbF.

### MiR-26b directly targets *MYB* 3′-UTR

**Fig. 6:**
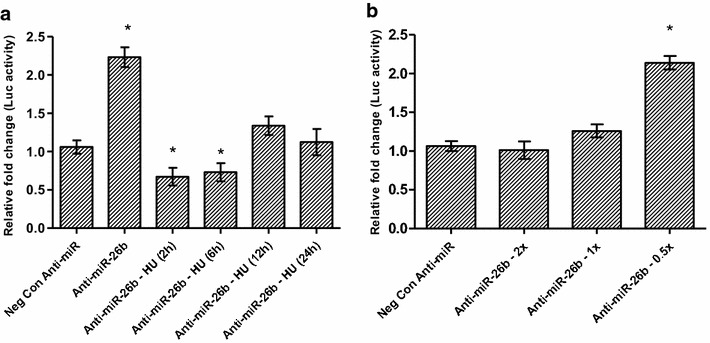
Time- and concentration-dependent luciferase activity in response to hydroxyurea treatment of anti-miR-26b transfected K562 cells. Inhibition of miR-26b results in a significant (p < 0.02) increase in luciferase activity and a sigmoidal pattern of action during a time course with HU treatment, with the highest luciferase activity observed after 12 h of treatment (**a**). A concentration gradient of anti-miR-26b demonstrates an inverse relationship between anti-miR26b concentration and *MYB*-3′-UTR luciferase activity. This unequivocally demonstrates interaction between miR-26b and the 3′-UTR of *MYB* (**b**). The likely implication of this result is that the miR-26b-induced translational inhibition of *MYB* (a known activator of *KLF*-*1*) could result in down-regulation of *KLF*-*1*, which is known to directly induce *BCL11A* expression. In sum, miR-26b could explain the observed up-regulation of γ-globin via a *MYB/KLF*-*1/BCL11A* pathway. * Significant difference, p < 0.05

Given the clear association between (i) HU and *MYB* expression; (ii) HU and our candidate miRNAs and (iii) the miRNAs and *MYB* expression patterns, dual-luciferase reporter assays were conducted using the pGL3-*MYB*-3′UTR to investigate the potential direct interactions between our candidate miRNAs and the *MYB*-3′-UTR (1191 base pairs). The amount of luciferase activity from the vector is directly proportional to the miRNAs occupancy of the UTR, which is known to induce mRNA transcript degradation via the RNA-induced silencing complex (RISC) [59]. There was a 2.2-fold increase in luciferase activity when cells were co-transfected with anti-miR-26b (Fig. 6a) and similarly, a time-dependent change in luciferase activity in response to HU treatment, with the highest luciferase activity observed after 12 h of HU treatment. Similarly, however less apparent, luciferase activity was observed with the transfection of anti-miR-151-3p, anti-miR-451 and anti-miR-494 (Additional file 5: Figure S5). To confirm that miR-26b directly interacts with the *MYB* 3′-UTR and initiates RISC-mediated mRNA degradation, there was gradual recovery of the luciferase activity with the decrease in concentration of the transfected anti-miR-26b (Fig. 6b). Therefore, at low anti-miR-26b concentration (2.5 nM), endogenous miR-26b is permitted to bind the *MYB* 3′-UTR, which would result in *MYB* mRNA degradation and therefore up-regulation of γ-globin expression.

## Discussion

Although HU has demonstrated significant clinical improvements in SCD patients, a complete understanding of the myriad of molecular mechanisms by which HU induces the disease-ameliorating HbF remain elusive [60]. This paper demonstrates the post-transcriptional effect of HU on critical regulators of γ-globin expression and associated miRNAs using two models; erythroid cells derived from umbilical cord blood CD34^+^ HSCs and K562 cells. Cord blood-derived CD34^+^ cells are an abundant, non-invasive and largely unutilised source of HSCs and a good model to investigate erythropoiesis [61, 62] and elucidate the molecular mechanisms of HbF production in response to therapeutic agents [63]. The volume of cord blood collected in this study was consistent with expected norms [64] although the average CD34^+^ cell yield was slightly lower, possibly due to our stringent cell separation method. Erythroid cells were successfully differentiated using an ex vivo single-phase expansion and differentiation protocol [65] with minor alterations to achieve optimal ex vivo expansion [66]. Given that HU is thought to induce γ-globin via perturbation of erythroid cell maturation [36, 67], in this study HSCs were differentiated to orthochromatophilic erythroblasts for 15 days. It has been previously demonstrated that cells treated with HU around day 6 and harvested at day 10 of differentiation consist mostly of basophilic and polychromatophilic normoblasts, comparable to untreated cultures at a similar stage [68]. In another study, investigating the effect of HU on *BCL11A*, *KLF*-*1* and *TAL*-*1* in bone marrow-derived basophilic erythroblasts, post terminal differentiation, there was no significant difference in the expression of the markers of late erythroid differentiation, CD235a and CD71, between HU treated and untreated cells [54]. Likewise in this study, the comparable levels of CD235a and CD71 between pairwise samples (HU+ and HU−) confirms that at the point of expression analyses, the paired samples were at a similar maturation stage (Additional file 1: Figure S1), and thus the erythroid cell maturation cannot account for the observed changes in expression. It is thus more likely that the observed induction of γ-globin is a result of the repression of *BCL11A, KLF*-*1* and *MYB*. Therefore, with further development of expansion and differentiation protocols, the use of cord blood stem cells can be extended to routine laboratory disease modelling and testing of therapeutic agents above and beyond the curative transplantation value of this biological material.

The WST-1 assay, a non-radioactive spectrophotometric quantification of cellular metabolic activity and proliferation was used to determine the optimal HU concentration and cellular incubation periods within a 24 h period given that HU is commonly prescribed to SCD patients as a daily oral drug [69]. The most apparent effects of HU on γ-globin, its critical regulators (*BCL11A*, *MYB*, *KLF*-*1*) and erythroid transcription factor*, GATA*-*1*, was between 6 and 12 h after 100 µM treatment, at which time cellular metabolic activity was sufficient to assess changes in gene expression.

Using paired analysis, we demonstrated a sevenfold (p < 0.003) up-regulation of γ-globin expression and corresponding down-regulation of *BCL11A* in both erythroid and K562 cells (Figs. 2, 3). *BCL11A* has been shown to be a critical negative regulator of γ-globin and fundamental to the ‘fetal switch’ from γ- to β-globin expression [58]. Furthermore, it has been successfully shown to be responsive to therapeutic manipulation for potential SCD treatment [65]. The time-dependent patterns of *BCL11A* and γ-globin gene expression are inversely related, with the most apparent effect at 6 h in both erythroid and K562 cells. Similarly, *KLF*-*1* was down-regulated in both cell models, corresponding to the repressed expression of *BCL11A* and β-globin gene (p < 0.04). This result is consistent with the function of *KLF*-*1* as an activator of *BCL11A* and its association with haplo-insufficiency-induced hereditary persistence of fetal hemoglobin [55]. *GATA*-*1* expression was similar to *KLF*-*1*, which is consistent with evidence that this principal erythroid transcription factor co-occupies the 5′ locus control regions and 3′ DNase I-hypersensitivity site of the β-globin gene cluster and associates with *BCL11A* in the Mi-2/nucleosome remodelling and deacetylases (NuRD) complex and therefore possibly critical in the repression of HbF production [58, 65, 70]. Down-regulation of *MYB* in both erythroid and K562 cells corresponded with induction of γ-globin expression and was inversely related to miR-26b as well as miR-15a and miR-16-1, which have been implicated in the elevation of HbF production via directly targeting the *MYB* transcription factor [45]. Although HU induced down-regulation of *BCL11A*; *KLF*-*1* and *MYB*, the effect on γ-globin expression was variable, which suggests that HU may interact with many genes upstream of γ-globin induction and also induce other post-transcriptional changes of gene expression through miRNAs. Recently similar effects of HU on *BCL11A* and *KLF*-*1* were demonstrated in late differentiation erythroblasts derived from bone marrow progenitors, also implicating *TAL*-*1* in the regulation of *BCL11A*; *KLF*-*1* via promoter-binding and *MYB* repression [54]. Furthermore, K562 cells were previously used to demonstrate the crucial role of *TAL*-*1* as the DNA-binding component of the *LDB*-*1* complex regulating the long-range looping of the globin gene cluster to allow interaction between the 5′-LCR and the β-globin gene [71]. Taken in sum with the results of the present study, HU induces γ-globin through numerous networks comprising *BCL11A, KLF*-*1, TAL*-*1* and *MYB*. The data of this study also demonstrated an association between miR-26b and γ-globin expression, which has previously been shown both at basal and MTD in HU-treated SCD patients [44]. There was a 4.5-fold up-regulation of miR-16-1 and miR-151-3p in K562 cells (Additional file 3: Figure S3), which was related to the most apparent induction of HbF at 6 h after HU treatment. A direct target of DNMT-1, miR-148a [72], was also up-regulated 6 h after HU treatment, which correlated to γ-globin induction. These findings suggest that the observed HbF production may also be through other post-transcriptional regulatory modalities such as methylation inhibition in the β-globin gene cluster.

**Fig. 7:**
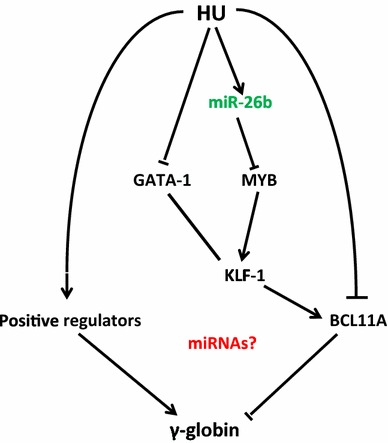
Hydroxyurea mechanisms of HbF induction: regulators and miRNAs mediated actions. Proposed HU-induced miRNAs-mediated mode of indirect HbF regulation through critical regulators (*MYB/KLF*-*1/BCL11A*) and possibly directly modulating HbF

The inhibition of the miRNAs, particularly miR-26b, unequivocally demonstrates a causative effect by HU on the induction of HbF through miRNA-mediated mechanisms. This is further supported by the near-complete transient knock-down of HbF when miR-26b and miR-494 are co-inhibited. The inhibition of miR-26b, miR-151-3p and miR-451 results in apparent decreases in HbF in comparison to all controls (inhibitor negative control; untransfected and HU treated cells). This effect is likely because miR-26b inhibits *MYB* translation, causing reduced *KLF*-*1* activation and thus lowering *BCL11A* expression, thereby increasing HbF production (Figs. 5, 6, 7). Therefore these data highlight the critical role played by potent negative regulators of HbF such as *MYB, KLF*-*1* and *BCL11A* and demonstrate that miRNAs provide a viable therapeutically responsive tier of HbF production for SCD treatment (Fig. 7), importantly while sparring the undruggable nature these transcription factors in non-erythroid cells [73]. Overall, the K562 cell experimental repeats were similar and although there was expected inter-individual variation in the degree and timing of up- and/or down-regulation of γ-globin and its key regulators in the erythroid model, the trends remained true and largely comparable. Despite the fact that several studies have and continue to utilise K562 as a model to investigate various components of γ-globin expression [39, 53, 74–77], it could be argued that K562 cells are not an ideal model for studying globin switching because of their bias expression of γ-globin. We however, saw this as an advantage in a number of ways: (1) the aim of this study was not to demonstrate the switching in expression of the globin genes (β to γ) but rather to use a stable cell line model in conjunction with cord blood-derived erythroid cells to demonstrate increases in γ-globin and decreases its regulators (*BCL11A, KLF*-*1* and *MYB*) in response to HU, and for this purpose, K562 cells would suffice as a model; (2) in choosing the whether to use miRNA mimics or anti-miRNAs (miR-inhibitors), the high expression of γ-globin in K562 was considered; if our hypothesis of HU-induced miRNA-mediated repression of *MYB* was correct, the use of mimics would not only introduce unnaturally high levels of miRNAs into this model (which we felt may in fact result in some artefactual results merely because of this unnatural state of aberrant concentration of miRNAs in the cells) but it would also be compounded by any co-treatments with HU in causing γ-globin expression. This rationale led to the choice of anti-miRNAs, which would go toward decreasing the already high levels of γ-globin in K562 (via *MYB*) and also provide an obvious opportunity of the rescue of miR-26b inhibition with HU co-treatment.

### Future work: implications for novel therapeutics in SCD

Future work regarding the post-transcriptional mechanisms regulating the expression of various trans-acting factors, such as *BCL11A, KLF*-*1* and *MYB*, could include experimental variations of the level of candidate miRNAs and examination of the effect on HbF levels and additional 3′-UTR luciferase assays on such regulators. The limitation on the number of primary erythroid cells could be overcome by the use of improved primary cell transfection protocols or alternative sources of HSCs such as commercially available HSCs or bone marrow aspirates. Future work will look to replicate these results in primary erythroid cells and investigate other candidate miRNAs for interactions with key regulators of γ-globin expression. Another limitation of the present work the “promiscuous” nature of HU, as it could influence many aspects of cellular functions and almost certainly alter the global cellular transcriptome. Therefore, future studies should continue to evaluate the in vivo impact of efficacious concentrations of HU on the erythroblast transcriptome and/or proteome, as well as the erythroid-specific micronome of SCD patients (before treatment and at MTD) as global analysis of these epigenetic mechanisms could highlight multiple components of this complex system and possibly yield alternative (possibly miRNA-based) therapeutic approaches to hemoglobinopathies [78]. This is likely not implausible as several clinical trials in other diseases have demonstrated a promising future for miRNA-based therapeutics such as the liposome-based human miR-34 mimic (MRX-34) miRNA-based drug against hepatocellular carcinoma (NCT01829971). MiRNAs have also been implicated in many cancers (colorectal, cervical, prostate, breast and lung) as either biomarkers and/or highlighted for potential therapeutic significance [79]. Closer to hemoglobinopathies, several miRNAs have been associated with specific phenotypes or key signalling pathways in SCD; miR-15a and miR-16-1 with hereditary persistence of HbF [53], miR-148a with DNMT-1 [72], miR-144 and tolerance of oxidative stress and anemia severity [52], a mouse model showing the correction of SCD via interference with the “fetal switch” [80, 81], in vivo association of miR-26b and miR-151-3p with HU treatment at MTD [44], miR-320 and down-regulation of CD71 during terminal differentiation of reticulocytes [51], miR-24 and inhibition of erythropoiesis via activin type I receptor *ALK4* [82], a suite of miRNAs including miR-451 in *Plasmodium falciparum* parasite interactions with erythrocytes [83] and now in this study, miR-26b direct interaction with *MYB* 3′-UTR and the rescue of miR-26b inhibition with HU treatment.

It is however noteworthy to consider the myriad of challenges in miRNAs-based therapies including site-specificity, delivery and treatment efficacies, off-targeting and side effects [84], undesired in vivo pharmacokinetics [85] and short half-life in peripheral blood before renal clearance [86]. These challenges may also be compounded by the rapid erythrocyte turnover and anemia in SCD, the heterogeneous multi-organ complications and the numerous genetic polymorphisms affecting disease severity, predisposition to specific phenotypes, the vast variation in response to HU treatment and the overall clinical course of the disease [60]. Although faced with many challenges, novel methodologies such as exosome-based delivery systems provide sufficient evidence in support of the continued efforts to develop improved systems of delivery and target-specificity, desired in vivo stability, reduced side- and off-target effects for miRNA-based therapeutic approaches [87–89].

## Conclusion

In the present article, the authors have demonstrated successful expansion and differentiation of umbilical cord blood-derived CD34^+^ HSCs into CD71^+^/CD235a^+^ erythroid cells and showed HU-mediated induction of γ-globin expression concomitant with the down-regulation of key negative regulators, *BCL11A*, *MYB* and *KLF*-*1*. Importantly, the experiments have demonstrated association between the induction of miR-15a and miR-16-1 and HU-mediated down-regulation of *MYB* as well as up-regulation of miR-148a, which targets DNMT-1. Furthermore, the data have shown the direct interaction between miR-26b and the *MYB* 3′-UTR, possible indirect modulation of *BCL11A* via *MYB* activation of *KLF*-*1*, all of which were concomitant with HbF production in both erythroid and K562 cells. The results of this study demonstrate the role of miRNAs in the modulation of HbF, directly and through critical regulators. The elucidation of the post-transcriptional regulation of HbF through miRNAs could incite investigation of therapeutic agents that would avoid global transcriptome changes but rather hone in on critical regulators with lineage-specific miRNA-mediated inhibition of negative regulation of HbF.

## Methods

### Umbilical cord blood

Umbilical cord blood was harvested during elective caesareans at Mowbray Maternity Hospital (Cape Town, South Africa) from healthy females of black African origins, free of the sickle cell mutation. Cord blood (90–140 ml) was collected in anticoagulant citrate phosphate dextrose adenine (CPDA-1)-containing bags (SSEM Mthembu, South Africa) and processed within 4 h of harvest.

### Isolation of CD34^+^ hematopoietic cells

Cord blood samples were diluted using Iscove’s Modified Dulbecco Medium (Sigma, SA), layered on Ficoll-Histopaque (1.077 g/ml) (Sigma, SA), centrifuged to collect the mononuclear cell layer (MNCL), then enriched for CD34^+^ hematopoietic stem cells (HSCs) using Dynabead magnetic separation technology according to manufacturer’s instructions (Life Technologies, SA).

### Flow cytometry

The purity of the Dynabead-selected HSCs and differentiated erythroid cells was determined using cell-surface antibodies and flow cytometry (FACSCalibur, BD Biosciences, USA). Briefly, 5 × 10^4^ HSCs were stained with phycoerythrin-conjugated CD34 (CD34-PE) and fluorescein isothiocyanate-conjugated CD45 (CD45-FITC) (BD Biosciences, USA) according to the manufacturer’s instructions and analysed. Subsequent to erythroid differentiation, markers of the derived-erythroid cells were analysed on day 15 using CD34-PE, CD45-APC, CD71-FITC and CD235a-PE (BD Biosciences, USA). The controls included in the flow cytometry experiments were as follows: (i) No cells (cell suspension fluid: 1x PBS), (ii) Cells only, (iii) Cells and primary antibody only, (iv) Cells and secondary antibody only. These were included to ensure specificity in antigen–antibody interactions in the experiments. During optimization, a separate cell line, RAMOS (RA-1 CRL1596, ATCC) was used to independently confirm the specificity of the primary antibodies (CD34, CD45, CD71 and CD235a) compared to K562 cells (using ATCC reference expression levels and those determined in our lab) and HSC-derived erythroid cells.

### Hematopoietic colony assay

Colony-forming unit-granulocyte, erythroid, monocyte, megakaryocyte (CFU-GEMM) hematopoietic colony assays were performed using 1 × 10^4^ HSCs seeded in semi-solid agar plates supplemented with 30 % fetal bovine serum (FBS) (Biochrom, Germany), 1 U/ml EPO, 200 ng/ml SCF, 10 ng/ml of both granulocyte-colony stimulating factor (G-CSF) and interleukin-3 (IL-3). The colonies were counted after 14 days in culture. After glutaraldehyde fixing, cultures were lifted, mounted on slides and stained with May-Grundwald and Giemsa stains then visualized under the microscope.

### Cell culture

CD34^+^ cells were cultured using a single-phase ex vivo expansion and differentiation protocol [53] with minor modifications. Briefly, cells were cultured in IMDM supplemented with 2 U/ml erythropoietin (EPO) (Sigma-Aldrich, Chemie GmbH Germany), 1 μM glucocorticoid dexamethasone (Sigma-Aldrich, Chemie GmbH Germany), 40 ng/ml insulin-like growth factor 1 (IGF-1) (Sigma-Aldrich, South Africa), 100 ng/ml stem cell factor (SCF) (Sigma-Aldrich, Chemie GmbH Germany) and 400 μg/ml holo-human transferrin (Sigma-Aldrich, Germany). The initial seeding density of 0.5−1 × 10^6^ cells/ml were expanded and differentiated to erythroid progenitors for 15 days, with ad hoc demi-population. Post-differentiation, flow cytometry was used to determine expression of CD71 and CD235a (Glycophorin-A). The human erythroleukaemia cell line K562 was cultured in IMDM initially supplemented with 10 % FBS and then 0.5 % in all subsequent experiments.

### Cell proliferation assay

The determine the optimal concentration and exposure time to HU, K562 cells treated and analysed using the WST-1 Cell Proliferation Assay (Roche) 2, 4, 6, 12 and 24 h after treatment. Briefly, the assay is a non-radioactive, spectrophotometric method to quantify cell proliferation and viability in response to HU treatment. A final concentration of 100 µM of HU was used in all subsequent experiments.

### Gene expression

Total RNA was isolated from both human erythroid and K562 cells using the Qiagen AllPrep DNA/RNA/miRNA Kit (Qiagen, USA) in accordance with the manufacturer’s instructions. Total RNA was used for mRNA and miRNA cDNA syntheses using the High-Capacity cDNA Reverse Transcription kit (Applied Biosystems, UK) and the miScript II Reverse Transcription Kit (Qiagen, USA), respectively. To investigate their suitability as internal control genes, the expressions of *RPS17*, *RSP20* and *GAPDH* were analysed in cells treated with HU. The expression of all three genes were relatively unaffected by the drug, and *RPS20* was chosen as the reference gene in all subsequent qPCR assays as it was least affected by the treatment. In order to explore a potential mechanism of post-transcriptional regulation, changes in expression of seven targeted miRNAs, previously shown to target *MYB* (miR-15a and miR-16-1) [53]; modified by HU (miR-148a, miR-151-3p and miR-494); associated with basal γ-globin expression (miR-26b) and with HbF at MTD (miR-451) [44] were also examined using miScript primer assays. Gene expression analysis was conducted using SYBR Green-based qPCR and individual primer assays for differential miRNA expression analysis and performed on the CFX96 Real-Time instrument (BioRad, USA). The controls used when the differential expression of miRNAs was investigated using commercially available single primer assays (Qiagen, USA) were samples with (i) No RNA, (ii) No reverse transcriptase and (iii) C. elegans spike-controls in cDNA synthesis and (iv) No cDNA control in the PC reaction. To use for normalization, the expressions of miRNAs SNORD68, SNORD95 and RNU6-2 were analysed for differential regulation by HU. Their expressions were relatively unaffected, and miRNA RNU6-2 was chosen as the reference gene for all subsequent assays due to its higher stability.

### Transfection

To establish whether there is a direct relationship between miRNAs and γ-globin expression upon treatment with HU, K562 cells were transfected with anti-miRNAs, individually as well as in various combinations. Cells were transfected with human miScript miRNAs inhibitors (anti-miRNAs) (Qiagen, USA); anti-miR-26b-5p, anti-miR-151a-3p, anti-miR-451a and anti-miR-494-3p using XtremeGene HP (Roche) for 24 h and treated with HU. To ensure that the transfection reagent (TR) did not affect HbF expression, no TR controls were included during miRNA and western blot assay optimization. In addition, the expression of genes was measured relative to cells transfected with the miScript inhibitor Negative Control (Qiagen, USA), which is a commercially available scrambled anti-miRNA that has no consensus with any known human miRNAs.

### Western blot

Cells were suspended in RIPA lysis buffer (150 Mm NaCl, 1 % Triton X100, 0.1 % SDS, 10 Mm Tris pH 7.5 and 1 % deoxycholate powder) and lysed overnight at −80 °C. The BCA kit (Pierce BCA Protein Assay kit, Thermo Scientific, USA) was used to quantify protein from total cell lysates and 15 μg of protein for each sample was loaded and separated on 15 % SDS-PAGE gels and transferred onto nitrocellulose membranes. Anti-fetal hemoglobin primary antibody was used at 1:1000 dilution (ab156584, Abcam, RSA), for loading control, 1:5000 dilution of anti-p38 (BioRad, USA) and 1:5000 dilution of goat anti-rabbit IgM horse radish peroxidase (HRP) conjugate secondary antibody (BioRad, USA) were used. Blots were developed using the Clarity ECL substrate (BioRad, USA).

### Luciferase assays

Dual-luciferase reporter assays (Promega, USA) were conducted using the pGL3-*MYB*-3′-UTR to investigate potential direct interactions between our candidate miRNAs and the *MYB* 3′-UTR (1191 base pairs) using the pGL3-*MYB*-3′UTR (Addgene, USA). The pGL3-*MYB*-3′-UTR vector contained a 1191 bp insert of the c-*MYB* 3′-UTR. The pGL3-basic vector was used as a control in the luciferase assays. The bacterial stab (growth strain DH5α) was streaked on Luria broth (LB) (Sigma-Aldrich, SA) plates and grown overnight at 37 °C with ampicillin antibiotic (100 µg/ml) and single colonies were inoculated in liquid cultures. The plasmids were recovered using the PureYield Plasmid Miniprep and Maxiprep kits (Promega, USA) and quantified using a nanodrop spectrophotometer (NanoDrop, USA). To confirm presence of the c-*MYB* 3′-UTR insert, restriction enzyme digests and agarose gel electrophoresis were performed. As further confirmation, the vectors were sequenced using RV-primer four (Molecular Cell Biology Department, University of Cape Town).

K562 cells were co-transfected with the luciferase reporter vectors and anti-miRNAs using XtremeGene HP DNA transfection reagent (Roche, USA) according to manufacturer’s instructions. All transfections included a pRL reporter vector with *Renilla* luciferase cDNA, to which all *MYB*-3′-UTR luciferase activity was normalized (*MYB*-3′-UTR: pRL-TK). Cells were lysed and the supernatant collected for analysis using the Luciferase assay reagent II (LARII) (Promega, USA) in a 96-well luciferase assay plate. The Glomax Multi-Detection system (Promega, USA) was used to measure the luminescence and the Stop & Glo reagent (Promega, USA) to quantify the *Renilla* luciferase signal for each well. The pGL3-basic vector was used as a baseline reference for all experimental wells with the results given as fold change of normalized luciferase signal over baseline.

The pGL3-Basic or Empty vector was used as a control to check if any of the co-transfected anti-miRNAs had background interaction with the backbone of the vector. There was no such interaction even when cells with the basic/empty vector were treated with HU. Luciferase activity was also measured in cells transfected with the pGL3-MYB-3′-UTR vector but not treated with HU in order to assess the effect of basal miRNAs on the 3′-UTR.

### Statistical analysis

For each gene expression experiment in K562 cells, three technical repeats (same experiment) and three independents experimental repeats were performed. Relative quantification using ΔCq values was calculated in accordance with the Pfaffl et al. method [90]. Statistical analysis was done using the student t test on ΔCq values and a p value of 0.05 or less was considered significant.

## Additional files

10.1186/s40169-016-0092-7Time dependent expression of markers of late erythroid differentiation (CD235a and CD71) in erythroid cells treated with HU. Expression of CD235a (A) and CD71 (B) during late erythroid differentiation show no significant differences between HU treated (+) and untreated (−) cells. The lack of significant changes in the expression of these markers is indicates that HU had minimal effect on the processes of erythroid differentiation, thus suggesting that the results, particularly the induction of γ-globin and repression of *BCL11A, KLF*-*1* and *MYB*, were not artefacts of stress erythropoiesis.
10.1186/s40169-016-0092-7Time-dependant effect of hydroxyurea on cellular metabolic activity. To determine the optimal exposure time to HU (100 μM previously determined to be the optimal concentration), K562 cells (8 × 10^4^ cells/100 μl) were plated in a 96-well dish in triplicates and treated with 100 μM hydroxyurea for 2, 4, 6, 12 and 24 h. Six (6) and 12 h exposure times were determined to be optimal as at this time point, the initial cytotoxic surge of HU had subsided and sufficient cells remained metabolically active to alter gene expression in response to the treatment. This determination was also supported by the fact that HU is prescribed to SCD patients as an oral pill taken daily, thus suggesting that the treatment takes most effect between 6 and 12 h after treatment.
10.1186/s40169-016-0092-7 Time-dependent effect of hydroxyurea on miRNAs expression in K562 cells. HU caused sigmoidal time-dependent changes in miRNAs expression in K562 cells with significant up-regulation of all miRNAs except miR-26b and miR494 (although not statistically significant, but also peaking in expression 6 h post treatment). MiR-16-1 and miR151-3p had a 4.5-fold up-regulation, which was associated with the most apparent induction of HbF at 6 h after HU treatment.
10.1186/s40169-016-0092-7Time dependent luciferase activity in response to HU treatment of anti-miR-151-3p; anti-miR-451 and anti-miR-494 transfected K562 cells. Luciferase activity was highest after 6 and/or 12 h of HU treatment in for all anti-miRNAs, demonstrating a sigmoidal pattern of luminescence. Although less apparent as compared to anti-miR-26b, the anti-miR-151-3p concentration gradient also showed a similar gradual increase in luciferase activity associated with the decrease in anti-miR-151-3p concentration (A and B). There was minimal data to suggest interaction between the *MYB*-3′-UTR and miR-451 and miR-494, in a 24-hour time course experiment (C and D).
10.1186/s40169-016-0092-7Concentration-dependent inhibition of miR-26b; miR-151-3p; miR-451 and miR-494 down-regulates HbF protein in K562 cells. HbF protein was reduced at higher concentration of all anti-miRNAs. This suggests that most miRNAs target negative regulators of HbF as their inhibition causes up-regulation of γ-globin expression. It is possible that like miR-26b, a suite of other HU-responsive miRNAs could modulate HbF production through regulation of positive and negative regulators of γ-globin.

## Abbreviations

SCD: sickle cell disease
FDA: Food and Drug Administration (USA)
HU: hydroxyurea
UTR: untranslated region
LCR: locus Control region
Hb: hemoglobin
Glu: glutamine
Val: valine
HbF: fetal hemoglobin
SSA: Sub-Saharan Africa
MTD: maximum tolerated dose
BFU-E: burst-forming unit-erythroid
CFU-GEMMs: colony-forming unit-granulocyte, erythroid, monocytes, megakaryocyte
CD: cluster of differentiation
HSCs: hematopoietic stem cells
RISC: rNA-induced silencing complex
NuRD: Mi2/nucleosome remodelling and deacetylases
PE: phycoerythrin
FITC: fluorescein isothiocyanate
APC: allophycocyanin
Suppl.: supplementary
