# Supplementary figures and images for: Hydroxyurea down-regulates BCL11A, KLF-1 and MYB through miRNA-mediated actions to induce γ-globin expression: implications for new therapeutic approaches of sickle cell disease
Source: Clin Transl Med. 2016 Apr 7;5:15. doi: 10.1186/s40169-016-0092-7 (PMC4824700; doi:10.1186/s40169-016-0092-7)

**A**

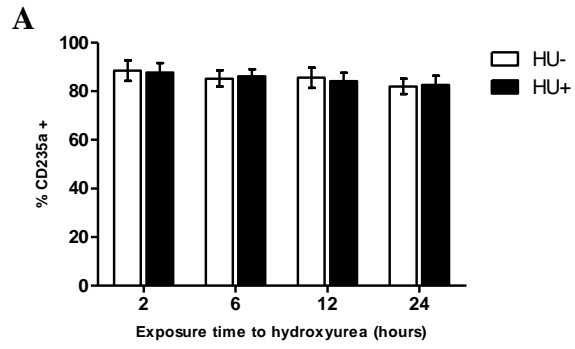

**B**

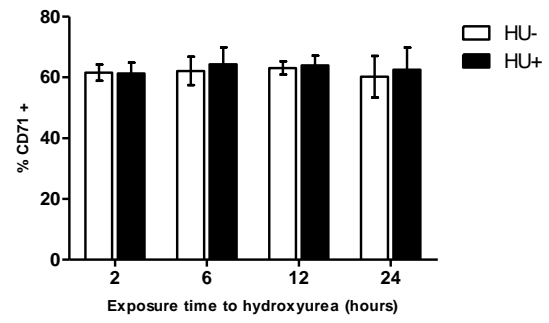

**Supplementary Figure 1**

Supplement: Supplementary file 1 — 10.1186/s40169-016-0092-7Time dependent expression of markers of late erythroid differentiation (CD235a and CD71) in erythroid cells treated with HU. Expression of CD235a (A) and CD71 (B) during late erythroid differentiation show no significant differences between HU treated (+) and untreated (−) cells. The lack of significant changes in the expression of these markers is indicates that HU had minimal effect on the processes of erythroid differentiation, thus suggesting that the results, particularly the induction of γ-globin and repression of BCL11A, KLF-1 and MYB, were not artefacts of stress erythropoiesis. [file 40169_2016_92_MOESM1_ESM.pdf]

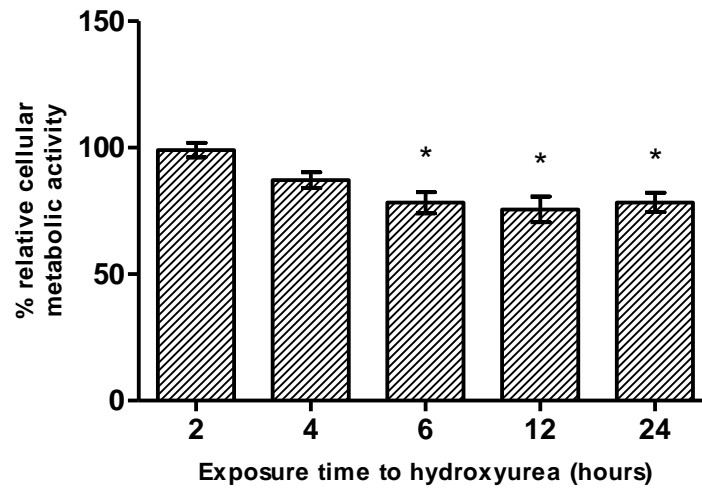

**Supplementary Figure 2**

Supplement: Supplementary file 2 — 10.1186/s40169-016-0092-7Time-dependant effect of hydroxyurea on cellular metabolic activity. To determine the optimal exposure time to HU (100 μM previously determined to be the optimal concentration), K562 cells (8 × 10^4^ cells/100 μl) were plated in a 96-well dish in triplicates and treated with 100 μM hydroxyurea for 2, 4, 6, 12 and 24 h. Six (6) and 12 h exposure times were determined to be optimal as at this time point, the initial cytotoxic surge of HU had subsided and sufficient cells remained metabolically active to alter gene expression in response to the treatment. This determination was also supported by the fact that HU is prescribed to SCD patients as an oral pill taken daily, thus suggesting that the treatment takes most effect between 6 and 12 h after treatment. [file 40169_2016_92_MOESM2_ESM.pdf]

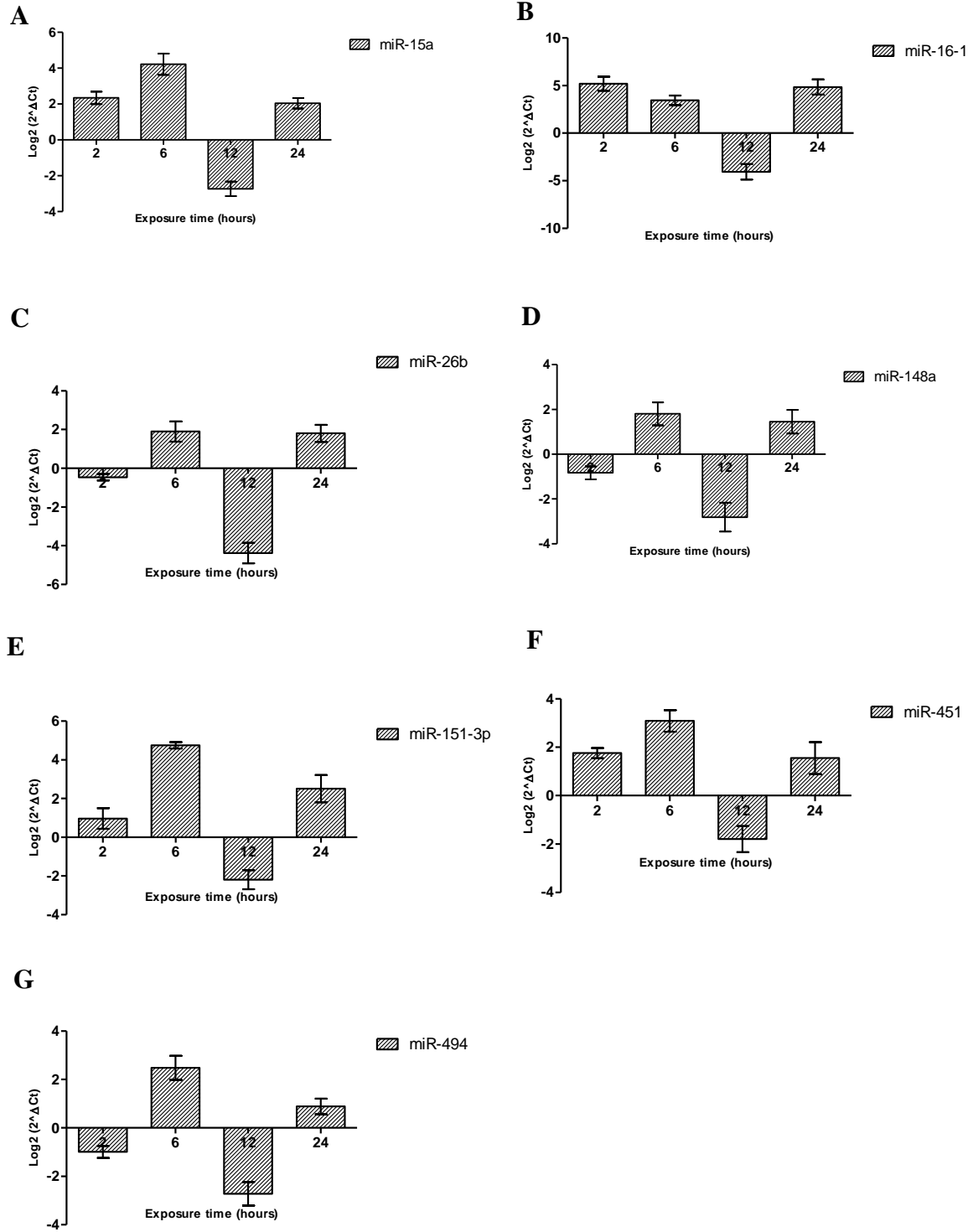

Supplementary Figure 3

Supplement: Supplementary file 3 — 10.1186/s40169-016-0092-7 Time-dependent effect of hydroxyurea on miRNAs expression in K562 cells. HU caused sigmoidal time-dependent changes in miRNAs expression in K562 cells with significant up-regulation of all miRNAs except miR-26b and miR494 (although not statistically significant, but also peaking in expression 6 h post treatment). MiR-16-1 and miR151-3p had a 4.5-fold up-regulation, which was associated with the most apparent induction of HbF at 6 h after HU treatment. [file 40169_2016_92_MOESM3_ESM.pdf]

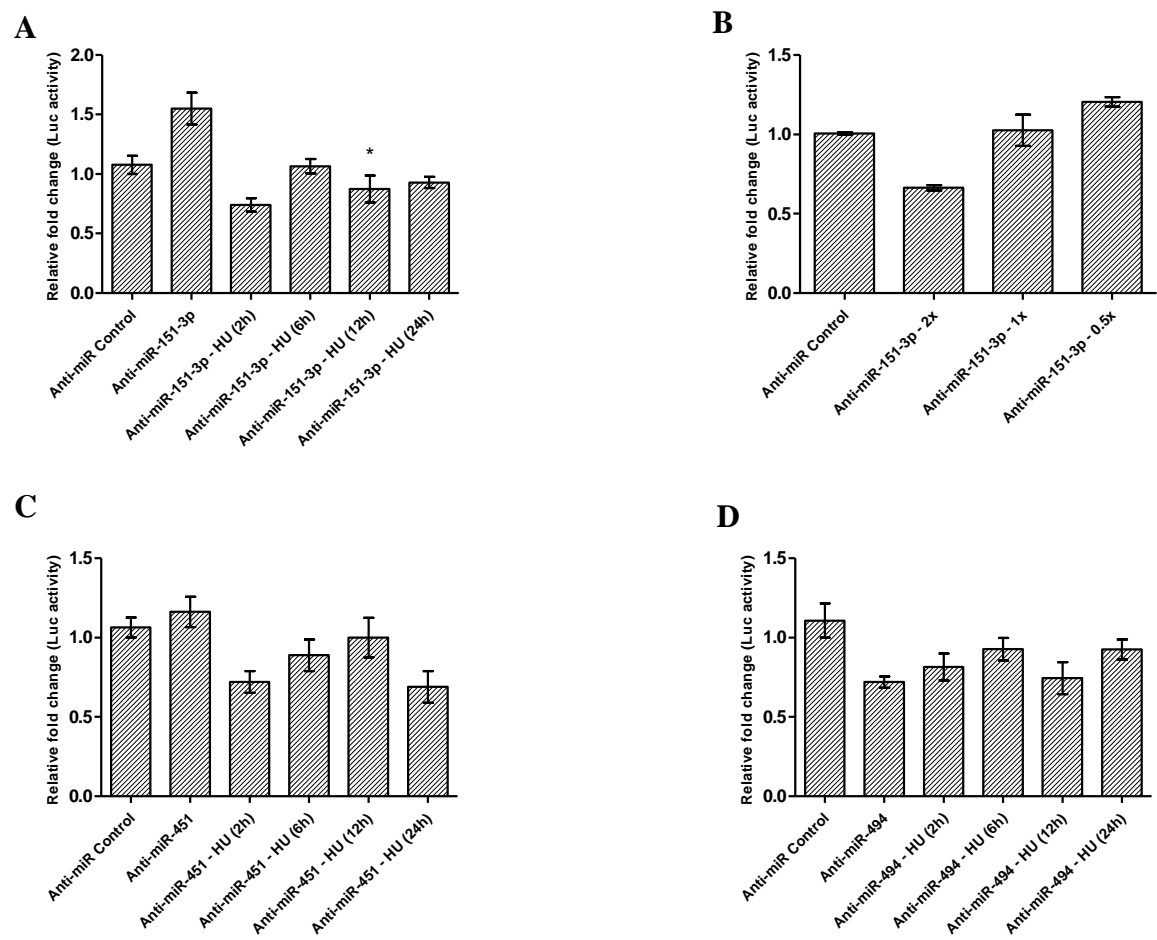

**Supplementary Figure 5**

Supplement: Supplementary file 4 — 10.1186/s40169-016-0092-7Time dependent luciferase activity in response to HU treatment of anti-miR-151-3p; anti-miR-451 and anti-miR-494 transfected K562 cells. Luciferase activity was highest after 6 and/or 12 h of HU treatment in for all anti-miRNAs, demonstrating a sigmoidal pattern of luminescence. Although less apparent as compared to anti-miR-26b, the anti-miR-151-3p concentration gradient also showed a similar gradual increase in luciferase activity associated with the decrease in anti-miR-151-3p concentration (A and B). There was minimal data to suggest interaction between the MYB-3′-UTR and miR-451 and miR-494, in a 24-hour time course experiment (C and D). [file 40169_2016_92_MOESM4_ESM.pdf]

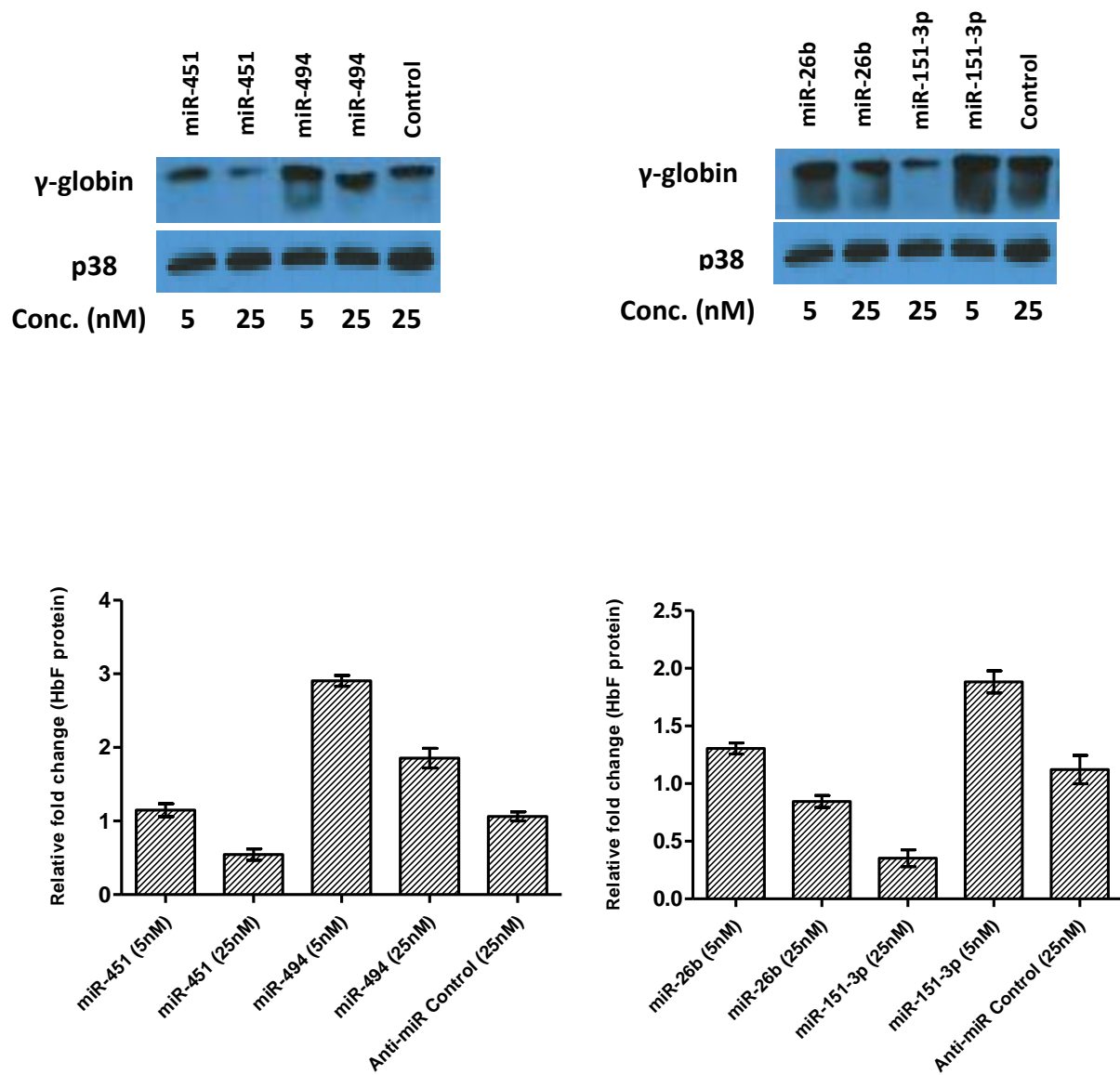

Supplementary Figure 4

Supplement: Supplementary file 5 — 10.1186/s40169-016-0092-7Concentration-dependent inhibition of miR-26b; miR-151-3p; miR-451 and miR-494 down-regulates HbF protein in K562 cells. HbF protein was reduced at higher concentration of all anti-miRNAs. This suggests that most miRNAs target negative regulators of HbF as their inhibition causes up-regulation of γ-globin expression. It is possible that like miR-26b, a suite of other HU-responsive miRNAs could modulate HbF production through regulation of positive and negative regulators of γ-globin. [file 40169_2016_92_MOESM5_ESM.pdf]
